# Tri-process model of interpersonal mindfulness: theoretical framework and study protocol

**DOI:** 10.3389/fpsyg.2023.1130959

**Published:** 2023-04-26

**Authors:** Bassam Khoury, Viktoriya Manova, Lena Adel, Guillaume Dumas, Michael Lifshitz, Rodrigo C. Vergara, Harmehr Sekhon, Soham Rej

**Affiliations:** ^1^Department of Educational and Counselling Psychology, McGill University, Montreal, QC, Canada; ^2^Integrated Program in Neuroscience, McGill University, Montreal, QC, Canada; ^3^Department of Psychiatry, University of Montreal, Montreal, QC, Canada; ^4^Department of Psychiatry, Jewish General Hospital, Lady Davis Institute, McGill University, Montreal, QC, Canada; ^5^Departamento de Kinesiología, Facultad de Artes y Educación Física, Universidad Metropolitana de Ciencias de la Educación, Santiago, Chile; ^6^McLean Hospital, Harvard University, Boston, MA, United States

**Keywords:** interpersonal, mindfulness, meditation, mindfulness-based stress reduction (MBSR), dyadic

## Abstract

According to the Center for Disease Control and Prevention, over 14% of the US population practice mindfulness meditation. The effects of mindfulness training on physical and mental health have been consistently documented, but its effects on interpersonal relationships are not yet fully understood or investigated. Interpersonal relationships play a crucial role in the wellbeing of individuals and society, and therefore, warrants further study. The aim of this paper is to present a tri-process theoretical model of interpersonal mindfulness and a study protocol to validate the proposed model. Specifically, according to the proposed model, mindfulness meditation training increases the self-awareness, self-regulation, and prosociality of those receiving the training, which ameliorates the quality of interpersonal interactions and the socioemotional support provided to other individuals. Finally, better socioemotional support increases the support receiver’s ability to regulate their emotions. Using a multiphasic longitudinal design involving 640 participants randomized into 480 dyads, the proposed protocol aims to validate the tri-process model and to investigate its mechanisms of actions. The proposed study has important theoretical and social implications and will allow devising new and more effective interpersonal mindfulness programs with applications in multiple fields.

## 1. Introduction

**FIGURE 1:**
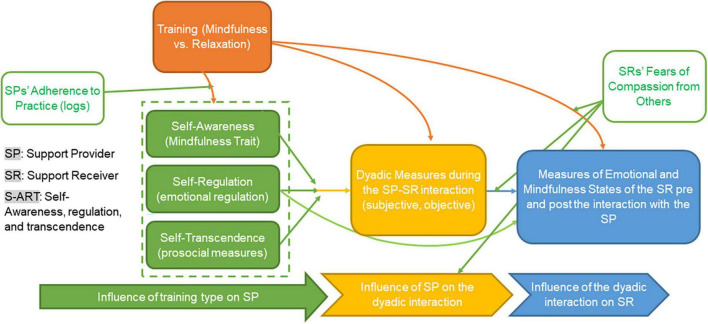
Schematic representation of the tri-process model of interpersonal mindfulness.

Recent studies suggest that mindfulness training not only has positive effects on those receiving the training, but also on the quality of their interpersonal relationships, and on the individuals who are part of these relationships. In fact, research suggests that mindfulness training has positive effects on self-awareness (e.g., mindfulness disposition), self-regulation (e.g., reduced stress and increase in emotional and cognitive regulation) and self-transcendence (measured as prosocial tendencies and behaviors among naive meditators rather than ego dissolution as is the case for expert meditators or Buddhist monks) (Vago and Silbersweig, 2012). Self-awareness, regulation, and transcendence, whether increased through sustained mindfulness training/practice or existed as innate traits/dispositions, impact interpersonal interactions through the direction and sustaining of attention toward others (e.g., active listening), increase in prosocial attitudes/behaviors (e.g., empathy, compassion, and perspective taking) and decrease of stress and reactive behaviors. Finally, positive interpersonal interactions have been shown to facilitate the emotional regulation of individuals who are part of the relationship, which might also free them cognitively and therefore, increase their ability to be mindful. This constitutes the tri-process model of interpersonal mindfulness (see Figure 1). According to this model interpersonal mindfulness is assumed to lead to better interpersonal interactions as previous studies indicated, however, this assumption remains to be empirically tested.

This paper aims to present the tri-process model of interpersonal mindfulness, its theoretical and empirical foundations, and to describe a study protocol, which tests the model and explores its mechanisms of action. Interpersonal mindfulness will be grounded in interpersonal interactions, specifically in the influence exerted by an individual with mindfulness training on another person (a stranger) during dyadic interactions. We call the individual with training a Support-Provider or SP (as they will provide socioemotional support) and the other a Support-Receiver or SR (as they will receive support from the SP). To empirically test this model, we are proposing the use of a randomized-controlled trial that aims to compare the effects of mindfulness training (MT) to an active control (relaxation training, RT) on (1) the SP receiving the training, (2) their interpersonal interactions with a stranger in a lab setting, and (3) the individual interacting with them and the mechanisms behind these effects. In detail, we aim to examine the effects of the type of training (MT vs. RT) on self-awareness (measured by mindfulness disposition), self-regulation (measured by stress and emotional/cognitive regulation) and self-transcendence (measured by prosocial tendencies/behaviors such as empathy, compassion, active listening, and perspective taking) of the SP receiving the training and providing support (Objective O1.1) and the moderators of these effects (e.g., adherence and quality of daily practice) (Objective O1.2). In addition, we aim to examine the effects of the type of training of the SP on the quality of their interpersonal relationship during a 15-min videotaped dyadic interaction with a stranger (SR) to whom they will provide socioemotional support (Objective O2.1) and the mediators of these effects (e.g., self-awareness, regulation, and transcendence of the SP; Objective O2.2). Moreover, we aim to investigate the effects of the type of training of the SP on changes in (a) affective and (b) mindfulness states of the individual receiving the support (SR) (Objective O3.1) and the mediators and moderators of these effects (e.g., quality of the SP/SR dyadic interactions as mediator and SR’s fears of compassion from others as potential moderator) (Objective O3.2).

### 1.1. Theoretical approach

There are two prevalent models for cultivating mindfulness in the context of a meditation practice; a 2500-year old Buddhist model and a contemporary 40-year-old model, that is heavily influenced by Kabat-Zinn (1990) Mindfulness-Based Stress Reduction (MBSR) program, which is an adaptation of specific Buddhist techniques intended for general stress reduction. Both models aim at reducing suffering and enhancing relationships (Vago and Silbersweig, 2012).

According to the Self-Awareness, Self-Regulation, and Self-Transcendence (S-ART) framework (S-ART; Vago and Silbersweig, 2012), which aims at synthesizing these two models, mindfulness is described as a mental training that is cultivated through meditation and that increases self-awareness, the ability to effectively modulate one’s emotions and behaviors (i.e., self-regulation), and creates a positive relationship between self and others that transcends self-focused needs and increases prosocial behaviors (i.e., self-transcendence). In this context, prosociality refers to behaviors, such as empathy, compassion, and perspective taking, that are intended to benefit others or decrease their distress (Jensen, 2016). The S-ART framework extends mindfulness from the intra-personal domain (e.g., enhancing self-awareness and reducing stress) to embracing its interpersonal and social effects (e.g., increasing prosocial behaviors). These components are also not mutually exclusive and may influence each other. For example, LeDoux and Brown (2017) higher-order theory of emotional consciousness asserts that self-awareness is crucial for emotional development and regulation. In this theory, an emotional state and a higher-order representation of that state (i.e., awareness) is needed to consciously experience the state or emotion and therefore regulate it. The second component of the S-ART model (i.e., self-regulation and specifically emotional and cognitive regulation) also has implications for both self and others given that modulating one’s emotional responses and behavioral reactions frees the resources required to accommodate someone else and help them regulate their emotional states (Finkel and Campbell, 2001).

Helping someone else regulate their emotions is designated as extrinsic interpersonal emotion regulation (Fischer and Manstead, 2016). Through Interpersonal Emotion Regulation (IER; Zaki and Williams, 2013), individuals (i.e., Support Providers, SPs) often attempt to regulate others’ emotions, through empathic, supportive, and prosocial behaviors. In the same process, the support receiver (SR) engages in intrinsic IER (i.e., aims to regulate their own emotions through the social emotional support provided by the SP; Williams et al., 2018). In addition, individuals actively seek out others after emotionally salient experiences (Taylor et al., 2004), and share their positive and negative emotions with them (Rimé, 2009). However, this requires an openness by the SR to receive social emotional support from others (Gilbert et al., 2011).

Inline with extrinsic IER, Coan’s Social Baseline Theory (SBT; Beckes and Coan, 2011; Coan and Maresh, 2014) posits that the human brain acts under the assumption that it is in a social environment. That is, proximity to others is the expected or baseline environment for humans. This is even more true when experiencing unpleasant emotions such as anxiety (Coan et al., 2006). In addition, Coan and colleagues argue that the employment of social emotion regulation is not only beneficial, but more effective and efficient because the brain is using less energy to regulate emotions (Coan et al., 2006; Beckes and Coan, 2011; Coan and Maresh, 2014). In support of the social nature of emotion regulation, it has been shown that the brain’s dorsolateral prefrontal cortex (dlPFC), which is important for self-regulation of emotions and is less active when an individual is around others who provide appropriate support (Eisenberger et al., 2007). The appropriateness of the support is mediated by the behaviors of the individual providing it (i.e., the SP; Mendes et al., 2003). In fact, individuals providing support (SPs) are perceived as more helpful when they display empathy and care toward others (Reis and Shaver, 1988), whereas negative emotional responses shown by Support Providers (SPs) were found to be related to higher stress and negative affect among those who were receiving support (i.e., SR; Rook, 2001; Kleiboer et al., 2007). Similarly, SBT suggests that when social support falls short of the baseline needed to return to a state of calm, it can lead to an increased need for personal resources, which often results in a decrease in emotion regulation capabilities (Diamond et al., 2008; Beckes and Coan, 2011). This indicates a central role of the self-regulation of Support Providers (SPs, as described in the S-ART framework) to provide effective support to others (SRs) and helping them to regulate their emotions.

Therefore, while the S-ART framework provides a conceptual model of how mindfulness training can impact mindfulness-meditation practitioners’ awareness, self-regulation, and prosociality, and through these mechanisms, their interpersonal interactions with others, IER and SBT provide theoretical explanations on how empathic and caring interpersonal interactions can help the individual on the receiving end of the interaction self-regulate. The integration of S-ART with IER and SBT, thus, provides the theoretical basis of the proposed tri-process model of interpersonal mindfulness (see Figure 1 for a schematic illustration of the tri-process model).

### 1.2. Literature review

#### 1.2.1. Effects of mindfulness training on the SP

In line with the S-ART framework (Vago and Silbersweig, 2012), randomized-controlled trials (RCTs) and systematic reviews/meta-analyses showed an increase of trait mindfulness and a reduction of stress following MT (e.g., MBSR; Khoury et al., 2013a,b, 2015). MT has also been implicated in successful self-regulation including attention and emotion regulation (Grossman et al., 2004; Ostafin et al., 2015; Tang et al., 2015). Improvements in emotion regulation associated with mindfulness training have been investigated through various approaches, including experimental, self-report studies, and measurement of peripheral physiology and neuroimaging (Hölzel et al., 2011). These studies have reported positive effects of MT on emotional regulation and processing (Zhang et al., 2019), such as decreased difficulties in emotion regulation (Robins et al., 2012). Consequently, lowered intensity of negative affect (Chambers et al., 2008), even following stressful situations (Weinstein et al., 2009), are reported to be associated with MT. For example, MBSR led to a decrease in negative emotions among individuals with social anxiety (Goldin and Gross, 2010) and an increased emotional regulation among a non-clinical population (Robins et al., 2012). MT increased acceptance of emotional experiences (Goldsmith et al., 2014), emotional awareness (Milicevic et al., 2016), emotional clarity (Cooper et al., 2018), and decreased impulsivity (Franco et al., 2016). Similarly, research showed that MBSR increased acceptance of emotional experiences (Goldsmith et al., 2014), decreased rumination (Deyo et al., 2009; Campbell et al., 2012), catastrophizing (Turner et al., 2016), and blaming others (Shahidi et al., 2017).

Beyond the effects on emotional regulation, MT has shown positive effects on prosocial tendencies and behaviors such as empathy, compassion, active listening, and perspective taking. In fact, MBSR increased empathy among medical (Shapiro et al., 1998), nursing (Beddoe and Murphy, 2004), and other healthcare students (Barbosa et al., 2013), as well as perspective taking among community samples (Birnie et al., 2010). Reviews and meta-analyses confirmed the positive effects of MBSR on perspective taking (Chiesa and Serretti, 2009) and on empathy and emotional competencies (Lamothe et al., 2016). MT yielded a positive increase in distress tolerance and compassion for others (Nila et al., 2016; Pommier et al., 2020). In a behavioral observation study, participants with MT gave their seat to a confederate showing signs of discomfort more frequently than participants in control groups (Lim et al., 2015). Otherwise, with the absence of quantitative experimental studies investigating the effects of MT on active listening, structural equation modeling suggested that trait mindfulness positively predicted active empathetic listening. In a recent study involving 137 participants in romantic relationships, trait mindfulness positively mediated the relationship between social skills and active empathic listening (Manusov et al., 2020).

Investigating the effects of an established mindfulness-based program (MBP) such as MBSR on a constellation of outcomes that can be related to interpersonal functioning, is the first objective of the proposed study (O1.1). In addition, most existing studies did not measure adherence to practice, which includes frequency, duration, quality, and type of practice and might be a viable moderator of the effects of MBPs (Del Re et al., 2013; Scott-Hamilton and Schutte, 2016; Parsons et al., 2017; Lacaille et al., 2018). Moreover, results from reviews suggested that increases in mindfulness following MBPs accounted partially for the amelioration on some of the above outcomes (e.g., stress and emotion regulation; Khoury et al., 2013a,b, 2015), however, to our knowledge no experimental study has quantified this relationship (which is objective O1.2).

#### 1.2.2. Effects of mindfulness training of the SP on the SP-SR interaction

The quality of social interactions depends on effective regulation of one’s emotions (Gross, 2002), and the latter requires skillfully responding to one’s own and others’ emotions (Zaki and Williams, 2013). Therefore, strategies that balance attentiveness to inner and outer affective events may be especially helpful. Recent evidence suggests that mindfulness may enhance emotion regulation in socioemotional contexts by enhancing conscious attention to one’s own and others’ actions and emotions (Wachs and Cordova, 2007; Quaglia et al., 2014). This is inline with the S-ART framework and extrinsic IER (Vago and Silbersweig, 2012; Zaki and Williams, 2013).

In fact, cross-sectional and experimental studies support these theoretical explanations demonstrating that trait mindfulness predicts greater attention to socioemotional stimuli (Creswell et al., 2007; Quaglia et al., 2016), as well as adaptive emotion regulation in challenging social situations (Brown et al., 2012). In a dyadic study among romantic partners, trait mindfulness facilitated relationship satisfaction through a heightened perception of the partners’ responsiveness (Adair et al., 2018). MT was found to increase emotion regulation (Zhang et al., 2019), compassion (Nila et al., 2016), empathy (Rimes and Wingrove, 2011), interpersonal wellbeing (Cohen and Miller, 2009), listening skills (Newsome et al., 2006), and working alliance (Schure et al., 2008; Christopher et al., 2011; Campbell and Christopher, 2012). MBSR training provided to both students and teachers led to improvements in social-emotional competencies among both groups in comparison with controls (Luong et al., 2019), MT for teachers showed similar positive effects on emotional support of teachers toward their students using a behavioral observational measure of classroom interactions (Jennings et al., 2017). As proposed in the S-ART framework, MT is key in the self-awareness, regulation, and transcendence of the individuals receiving the training and these mechanisms are central in interpersonal functioning (Wei et al., 2005; Rawn and Vohs, 2006). However, no study has directly investigated the effects of mindfulness training on the quality of dyadic interactions using objective and subjective measures (O2.1). In addition, no study delineated the potential mediators of these effects (e.g., self-awareness of the SP) (O2.2).

#### 1.2.3. Effects of a mindfulness training of the SP on the SR

As discussed above, MT of the SP has positive effects on their self-awareness, regulation, and transcendence and in consequence, potentially on SP-SR interactions. These positive effects can be then transferred to the SR. This transfer can be understood through the intrinsic IER (Zaki and Williams, 2013) and SBT (Beckes and Coan, 2011; Coan and Maresh, 2014) lenses. Even though very little research has examined the extra-personal effects of MT, recent studies provided preliminary support for these theoretical foundations. For example, a study of 2,237 parents found that trait mindfulness of the parents was negatively associated with internalizing and externalizing problems in their children and that these associations were mediated by supportive parenting practices (Han et al., 2019). Similarly, studies found positive effects among children whose parents received a MBP (Singh et al., 2006, 2010; Duncan et al., 2009; Bögels et al., 2014; Coatsworth et al., 2015; Turpyn and Chaplin, 2016). In a recent dyadic study with romantic partners, one member of each dyad was randomly assigned to meditate daily for 15 min; the other member did not meditate. The study followed an A B A B design to compare non-meditation with meditation phases (May et al., 2020). Results suggested meditation was associated with decreased negative affect (NA), increased positive affect (PA), and higher mindfulness disposition. Results further demonstrated that the NA of non-meditating partners decreased during the weeks that their partner meditated. This study indicates that a short daily meditation in novice meditators can decrease the NA of relationship partners (May et al., 2020). An earlier study showed that the quality of dyadic interactions was associated with the PA of both members of the dyad (Berry and Hansen, 1996).

Recent research suggested that the SR’s acceptance (vs. fear) of support from others had an impact on the effectiveness of such support (Gilbert et al., 2011; Kirby et al., 2019). To our knowledge no study examined the impact of MT of one member of a dyad on the other’s affective state among unrelated dyads [O3.1(a)]. In addition, the mechanisms mediating and moderating these effects remain to be investigated (O3.2). Moreover, the SR’s affective states may impact their mindfulness state. For example, in a cross-sectional study, levels of state-mindfulness were positively related to levels of PA (Jislin-Goldberg et al., 2012). Thus, there is a potential transactional relationship between SR’s mindfulness state and PA. In fact, several studies have documented that experimentally induced positive emotions widen the scope of attention, and attentional selection (Fredrickson and Branigan, 2005; Wadlinger and Isaacowitz, 2006; Rowe et al., 2007) in comparison with negative and neutral states. Therefore, in theory, the development of a mindful state may drive the development of greater PA (Blanke et al., 2018), and greater PA may promote the development of a mindful state. Thus, it is highly warranted to examine the impact of a supportive dyadic interaction on the SR’s mindfulness state [O3.1(b)].

### 1.3. Main hypotheses

The following are the principal hypotheses of the study, and the numbering of the hypotheses is linked to the numbering of the objectives (e.g., H1.1 designates the hypothesis linked to the objective O1.1, Hbase designates the hypothesis at baseline, i.e., before randomization and the experimental procedure).

(H1.1) (a) Measures pertaining to S-ART will be significantly higher in SPs who receive MT compared to those who receive RT and thus, at post-training and at follow-up. The only measure that we hypothesize to be equally lower for both groups (at both time points) is stress. (b) Within-groups effects (i.e., among the three time points) will be significant for all the outcomes for the MT group, and only for stress for RT.

(H1.2) (a) The effects of MT on S-ART measures will be moderated by the adherence of the SPs to the practice at both time points (i.e., post-training and follow-up). (b) The effects of RT on stress will be moderated by the SPs’ adherence to practice.

(H2.1) (a) Objective and subjective measures pertaining to the quality of the SP-SR interactions will be significantly higher in dyads with a SP who received MT compared to dyads with a SP who received RT at both time points (i.e., post-training and follow-up). (b) Within-groups effects (across time) will be only significant for the dyads with an SP who received MT.

(H2.2) The effects of MT of the SPs on the quality of SP-SR interactions (measured subjectively and objectively) will be mediated by the increase on S-ART of the SPs at both times points (i.e., post-training and follow-up).

(H3.1) SRs who interacted with SPs who received MT training will show significantly (a) higher positive emotional states, lower negative emotional states and (b) higher mindfulness states following their interaction compared to those who interacted with SPs who received RT and thus, at both time points (i.e., post-training and follow-up). (c) Within-groups effects will be only significant for the SR who interacted with an SP with MT training.

(H3.2) The effects of MT of the SP on the emotional and mindfulness states of the SR will be mediated by the increase in the quality of the SP-SR interaction and moderated by SRs’ Fears of Compassion from Others (i.e., higher fears of compassion will reduce the mediation effects) at both time points (i.e., post-training and follow-up).

(Hbase) (a) At baseline, the S-ART measures of the SPs will be associated with the quality of SP-SR interactions (as measured objectively and subjectively) and with the emotional and mindful state of the SR and (b) the association between the S-ART of the SPs and the emotional/mindful states of the SR will be mediated by the quality of SP-SR interactions and moderated by Fears of Compassion from Others.

## 2. Methods and design

### 2.1. Participants

We aim to recruit 640 adult community members (18–35 years old, 50% female) through advertising on social media. The sample size was based on detecting significant differences among the groups (i.e., between SPs with MT vs. SPs with RT), assuming a moderate effect (Khoury et al., 2013a,^2015^), a power of 0.8 and an alpha of 0.05 (Cohen, 1977) and the expectation 15 to 20% attrition rate. We will exclude participants who: (1) have a regular meditation practice; (2) have already participated in a formal mindfulness training (such as MBSR or a meditation retreat); or (3) had a social support skills training (e.g., counselors, psychologists, social workers, or other medical personnel).

### 2.2. Experimental manipulations

#### 2.2.1. Mood induction

The mood induction will last for about 15 min and will consist of asking participants to recall a difficult life event during which they experienced intense negative emotions (e.g., sadness, anxiety, or anger) and to write on paper the information regarding this event (e.g., age at the time of the event, place of the event, and individuals present during the event). Similar autobiographical mood inductions were used previously and were shown to be very effective (Baker and Guttfreund, 1993; Smith et al., 1997; Mayberg et al., 1999). For ethical considerations and to provide participants with a choice and a sense of agency and protect them from any negative effects, they will be informed in advance about the procedure, and provided with the choice to stop the experiment at any time (i.e., before, during, or after the mood induction, as well as before or during the discussion of the event with the other member of the dyad). They will also provide with a list of psychological resources if needed.

#### 2.2.2. Mindfulness training (MT)

In the absence of a validated protocol for interpersonal mindfulness in a general context, that is, outside parenting (Kabat-Zinn and Kabat-Zinn, 1997), couple (Carson et al., 2004), or counseling (Cohen and Miller, 2009), MBSR remains the most used and validated protocol for the general population (Khoury et al., 2015). MBSR is an 8-week, 2.5 h weekly session program that comprises different types of meditations (sitting, walking, eating) in addition to yoga, psychoeducation, group discussion, a half-day meditation retreat, and at home practices (40 to 45 min 6 days weekly). Therefore, MBSR requires participants to commit to 2.5 to 3 h each week and 45 min of mindfulness practice each day for 8 weeks. This can impact adherence to the program. In fact, adherence to standard MBSR is approximately 85% among highly motivated participants, whereas among a student population, the adherence rate falls to 70% (Williams et al., 2001). Taking that into consideration, we will implement a shortened version of MBSR, called MBSR-ld (light). In MBSR-ld, the duration of weekly meetings is reduced to 1 h, the daily practice is also reduced to 20 min (Klatt et al., 2009). Studies showed significant improvement in mindfulness disposition following MBSR-ld (Bergen-Cico et al., 2013), comparable effects of MBSR-ld to the original protocol (Klatt et al., 2009), and lack of support of the significant effects of the number of in-class hours on MBSR outcomes (Carmody and Baer, 2009). Similarly, in a recent RCT, low-dose mindfulness-based intervention led to significant increase in trait mindfulness, self-efficacy, body awareness, and reappraisal (Karing and Beelmann, 2021). Each group will include 6 to 8 participants. Sessions will be facilitated by two Doctoral trainees who received formal MBSR training. To ensure the quality of the delivery of the program the trainees will be supervised by a registered clinical psychologist, certified in MBSR.

#### 2.2.3. Relaxation training (RT)

The relaxation training (RT) is an active control, which is similar in structure and duration to MT. Sessions will be facilitated by 2 Doctorate level trainee counselors. Each session will include 1 h that integrates techniques of autogenic and progressive muscle relaxation, guided imagery, psychoeducation, and group discussion. Participants will be asked to practice these exercises at home for 20 min 6 days weekly using pre-recorded audio files. Similar protocols were used as a control group for mindfulness training (Jain et al., 2007).

### 2.3. Randomization and allocation concealment

**FIGURE 2:**
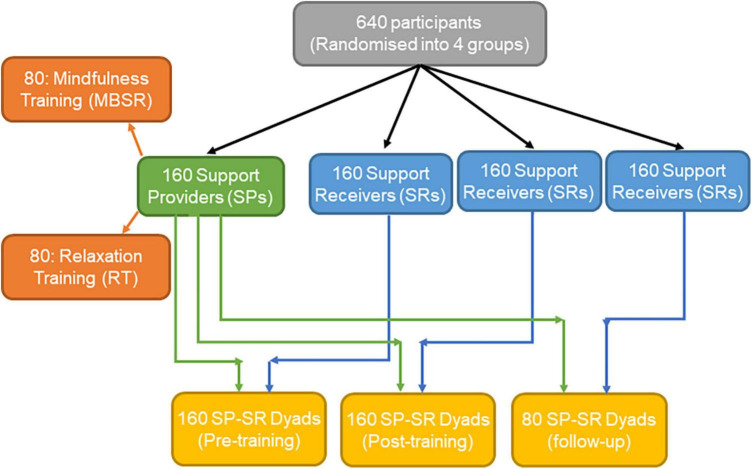
Schematic representation of the randomization process.

A total of 640 participants will be randomly divided into four equal groups (of 160 each) using a computer-based algorithm administered by a third party to ensure concealment. One group will be designated as SPs while the other three will be designated as SRs. Thus, for each SP, three dyads with different SRs will be formed using a computer algorithm to ensure randomization, i.e., (SP, SR1) to assess the quality of the dyadic interaction pre-training, (SP, SR2), to assess the quality of the dyadic interaction post-training, and (SP, SR3) to assess the quality of the dyadic interaction at follow-up (total of 3 × 160 = 480 dyads; see Figure 2). The experimental design, allocation procedure, and analyses are intended to minimize the effects of the dyad (i.e., unique combination of SP/SR in a dyad) vs. the SP and SR effects on the measured outcomes. In addition, according to the power analysis using Monte Carlo simulation (Schoemann et al., 2014), which is appropriate for dyadic research (Ledermann et al., 2022), 80 dyads per group should be sufficient (powered enough) to detect moderate effects (i.e., changes between baseline, post, and follow-up time points), and thus, beyond the variability between the dyads (e.g., personalities and other personal/interpersonal characteristics).

### 2.4. Materials and measures

Sociodemographic characteristics of all participants (SP and SR) such as age, race, ethnicity, gender, occupation, and socioeconomic status will be measured using a sociodemographic questionnaire that will be developed for the study. The effects of the training on the SP will be assessed based on the S-ART framework using:

(1) Self-Awareness measures, including trait mindfulness using the Mindful Attention and Awareness Scale (MAAS; Brown and Ryan, 2003), with good reliability/validity (Osman et al., 2016) and Breath Counting Task (BCT) as a behavioral measurement of mindfulness (Levinson et al., 2014). We elected to use the MAAS as it directly measures self-awareness, which is a central element in the proposed model. In addition, the use of beath counting adds an objective behavioral measure to mindfulness and has been shown to be highly sensitive to mindfulness training (Isbel et al., 2020).

(2) Self-Regulation measures, including stress using Perceived Stress Scale (PSS; Cohen et al., 1983), good psychometric properties (Lee, 2012) and emotional regulation using Difficulties in Emotion Regulation Scales (DERS; Gratz and Roemer, 2004) and the Cognitive Emotional Regulation Questionnaire (CERQ; Garnefski et al., 2001). While the DERS focuses on regulation of negative feelings, the CERQ assesses cognitive strategies in emotional regulation, with both scales having shown strong psychometric properties (Garnefski and Kraaij, 2007; Kaufman et al., 2016).

(3) Self-Transcendence (prosocial) measures, including empathy and perspective taking using the Interpersonal Reactivity Index, (IRI; Davis, 1980), good psychometric properties (Davis, 1983), compassion for others using the Compassion Scale (CS; Pommier et al., 2020), and active listening using the Active-Empathic Listening Scale, (AELS; Bodie, 2011), which includes sensing, processing and responding.

In addition, the effects of the training on the SPs’ interpersonal mindfulness skills will be assessed using our recently developed and validated measure of interpersonal mindfulness, Interpersonal Mindfulness Questionnaire (IMQ; Khoury et al., 2022), which is based on Varela’s work (Varela et al., 1991, 2016) and the theory of embodiment (Khoury et al., 2017, 2019, 2020; Khoury, 2018) and include four sets of global trainable skills (detachment from automatic thoughts, body anchored presence, attention to and awareness of the other person, and empathic responding; Khoury et al., 2023). These skills were shown to highly relate to interpersonal functioning and were sensitive to meditation practice or mindfulness training (Khoury et al., 2022).

Daily logs will be used to measure adherence to and quality of home practice (during the 8-week training and the 3 months follow-up). In order to reduce the time for participants and increase feasibility, we will use the short forms (SF) of the scales when available (e.g., CERQ-SF; Garnefski and Kraaij, 2006; DERS-SF; Kaufman et al., 2016).

The effects of the training on the SP-SR interaction (O2.1/O2.2) will be assessed subjectively by the SRs using the Multidimensional Evaluation of Enacted Social Support (MEESS; Goldsmith et al., 2000), which includes three dimensions: *helpful*, *supportive*, and *sensitive* (Goldsmith and Fitch, 1997; Goldsmith et al., 2000; Goldsmith, 2016; Goldsmith and Griscom, 2017). While helpfulness focuses on providing assistance, or perspective (Dunkel-Schetter and Bennett, 1990; Goldsmith, 1992, 1994; Cutrona and Suhr, 1994), supportiveness refers to validation (non-judging or criticizing) (Winstead et al., 1992; Cutrona and Suhr, 1994) and sensitivity relates to the emotional support (Burleson, 1994; Burleson and Goldsmith, 1996). The scale is composed of 12 semantic items scaled on seven points and demonstrated high reliability and construct validity (Goldsmith et al., 2000). The MEESS was developed using student/community samples and has since been used in a variety of populations and situations (Goldsmith and Griscom, 2017), making it highly suitable for this study. To rate the quality of the dyadic interaction objectively, we will use the Behavioral Observational Measure of Dyadic Interactions (BOMDI; Cuperman and Ickes, 2009), which assesses verbal and non-verbal behaviors of the SP during their interaction with the SR. The BOMDI comprises seven verbal and nine non-verbal items. Verbal items include for example, the number of verbal acknowledgments displayed by the SP. Non-verbal items include for example, head nods, facial expressions, and eye contact. These measures assess the level of empathic listening and perspective taking of the SP during the interaction. Inter-rater reliability is high (Cuperman and Ickes, 2009). To ensure better reliability, two independent trained research assistants will code the dyadic interaction according to the BOMDI. Differences of two points or more between the raters will be discussed and if consensus is not reached, the primary investigator will be contacted.

As suggested by IER and SBT, attentive, empathic, and caring interpersonal interactions can help the SR to better self-regulate and such a regulation process is economic (i.e., requires less cognitive and emotional resources), therefore automatically triggered without the conscious awareness of the individual on the receiving end (Eisenberger et al., 2007). This is even more true following a negative emotional state, such as following the negative mood induction. SBT suggests that this process allows the support receiver (SR) to return to a baseline, which in our experimental paradigm, is a return to an emotional/mindful state similar to that before the mood induction. Therefore, the effects of mindfulness training of the SP on the SR (O3.1/O3.2) will be assessed using the Positive and Negative Affect Schedule (PANAS; Watson et al., 1988), which comprises 20 items, 10 measuring PA (e.g., excited, internal consistency = 0.89), and 10 NA (e.g., upset, internal consistency = 0.85). The PANAS was designed to measure affect in various contexts such as at present, and in the past, and demonstrated high construct validity (Crawford and Henry, 2004). Thus, it can be used to measure fluctuations in emotions (Tran, 2013), making it highly suitable for the current study. In addition, state mindfulness of the SR will be measured using the Mindful Attention Awareness Scale-State (MAAS-State; Brown and Ryan, 2003), which includes only five items and can be used in a non-meditative context, making it suitable to measure fluctuations in mindfulness state in the current context. To control for the response of SRs to the social support received from the SPs, we will use the Fears of Compassion from Others (FOCO; Gilbert et al., 2011). Finally, to control for Social Desirability we will use Marlowe-Crowne Social Desirability Scale (SDS; Crowne and Marlowe, 1960) for the SP and SR. Measures will be completed either using a paper-based format or on a tablet depending upon the preference of the participants. See Table 1 for a summary of the sequence of administering the measures. To film and record the dyadic (SP-SR) interactions, a high-definition 360-degree camera (1080P HD 360°Camera) with a built-in recording system will be used. The experiment will take place at the first author’s lab at McGill University.

**TABLE 1:** Sequence of administering the measures during the experiment.

Outcome (variable)	Variable type	Dyadic interaction Part I, III and IV of the experiment				Mindfulness/Relaxation training Part II of the experiment			
		Pre-induction	Post-induction	During interaction	Post-interaction	Pre-training	During training	Post-training	3-months follow-up
Trait mindfulness	Dep/Cntrl					SP		SP	SP
Perceived stress	Dependent					SP		SP	SP
Emotional regulation	Dependent					SP		SP	SP
Empathy	Dependent					SP		SP	SP
Compassion–Others	Dependent					SP		SP	SP
Active listening	Dependent					SP		SP	SP
Perspective taking	Dependent					SP		SP	SP
Interpersonal mindfulness	Dependent					SP		SP	SP
Behavior observation	Dependent			SP-SR					
Enacted social Support	Dependent				SR				
Adherence to practice	Control						SP		
Emotional experience	Cntrl/Dep.	SR	SR		SR				
State mindfulness	Dependent	SR	SR		SR				
Fears of compassion	Control	SR							
Social desirability	Control	SR				SP			

Dep, dependent; Cntrl, control; SP, support provider; SR, support receiver.

### 2.5. Experimental procedure

#### 2.5.1. Experiment–Part I; pre-training

A total of 160 (SP, SR1) dyads will be invited to the lab. Upon arriving to the lab, members of each dyad will meet separately with two different research assistants (RAs). After signing a consent form, providing basic socio-demographic data, and filling-in the SDS, the SR will be asked to complete the FOCO (Gilbert et al., 2011), PANAS (Watson et al., 1988), and the MAAS-State (Brown and Ryan, 2003). Afterward, the SR will receive the 15-min mood induction while the SP will wait in a separate room doing an activity of their choice (e.g., reading popular magazines). Then, the SR will be asked to complete the PANAS and MAAS-State for a second time (as a check of the induction). Afterward, the SP and SR will be introduced to each other, the SR will be asked to discuss with the SP the event (which was part of the induction), and the SP will be asked to provide support to the SR as best they can during a 15-min videotaped interaction. The interaction will occur face-to-face, will be recoded, and the video will be rated by two trained graduate students according to the BOMDI (Cuperman and Ickes, 2009). Both the SP and SR will also be blind to the objectives of the study. Following the interaction, the SR will be asked again to complete the PANAS, MAAS-State, and the MEESS (Goldsmith et al., 2000). At the end of this part of the experiment, the SR will be debriefed about the study and its objectives.

#### 2.5.2. Experiment–Part II; mindfulness/relaxation training

SPs (total of 160) will be asked to complete measures of mindfulness, perceived stress, emotion regulation, empathy, compassion for others, active listening, perspective taking and interpersonal mindfulness. Then, they will be randomly and equally divided into two groups using a computer algorithm to ensure randomization and concealment. Half of the SPs (80) will receive the MT (MBSR-ld) and the other half will receive the RT. Participants in both groups will be asked to complete the daily logs during the 8 weeks. After the training, the same measures will be administered to the SPs.

#### 2.5.3. Experiment–Part III; post-training

This part will take place after the end of the training and is similar to Part I, the only difference is that SR1 will be replaced by SR2 in the dyads. In addition, only the SR will complete the consent form and provide socio-demo/social desirability data, as SPs already did that in Part I.

#### 2.5.4. Experiment–Part IV; follow-up

This part takes place 3 months after the end of the training and is similar to Part III (with the only difference being that SR2 will be replaced by SR3 in the dyads). SPs will be asked to complete the same measures (mindfulness disposition, perceived stress, emotion regulation, empathy, compassion to others, active empathic listening, perspective taking, and interpersonal mindfulness skills). At the end of the experiment, both SPs and SRs will be debriefed about the study and its objectives.

### 2.6. Timeline

The recruitment of participants will be divided into four cohorts (one cohort per year of 160, 40 SPs, and 120 SRs). The four parts of the experiment (i.e., Part-I, Part-II, Part-III, and Part-IV), will be repeated for each of the four cohorts. During each year (four in total), 40 SPs will be receiving training [MT(20), RT(20)]. The SPs receiving MBSR or RT will be divided into groups of 6 to 8 participants, and, a total of six groups will be conducted each year. We will conduct two groups (one MBSR, one RT) simultaneously during the fall, winter, and summer academic semesters of each year.

## 3. Data analyses strategies

To ensure data reliability, self-reported data will be inspected for normality and skewness and outliers will be eliminated. Interrater reliability for the behavior observation data will be computed. An interrater coefficient of 0.95 or above is expected.

To test H1.1, H2.1, and H3.1, we will perform a mixed (2 groups × 3 time-points) multivariate analysis of covariance (MANCOVA) controlling for social desirability (and for Fears of Compassion from Others for H3.1). MANOVA/MANCOVA allow to reduce Type-I errors (i.e., rejecting the null-hypothesis when it is true, correcting therefore for multiple comparisons) when testing changes in multiple dependent variables. To test H1.2, we will use a regression analysis. To test H2.2 and H3.2, we will use a path analysis. To test associations and meditation/moderation at baseline (Hbase), we will use correlations and PROCESS macro with 5,000 bootstrapped/bias-corrected confidence intervals (Hayes, 2018). Structural Equation Modeling (Klem, 2000) may be used as well if the power is sufficient.

Regarding the dyadic data, we will use the Actor-Partner-Interdependence-Model (APIM; Kenny and Judd, 1986; Kenny and Malloy, 1988; Griffin and Gonzalez, 1995; Kenny, 1996; Kenny et al., 2020) and APIM-M (M for mediation) as we cannot assume independence between the members of the dyad (Gonzalez and Griffin, 2012). APIM/M allows us to test the interactions between the SP and the SR scores on a given predictor (Kenny et al., 2020). To calculate power for the APIM/M, we used a Monte Carlo simulation (Schoemann et al., 2014), which is applicable for APIM/M (Ledermann et al., 2022). Results suggested that 80 dyads per group should be sufficient (powered enough) to detect moderate effects (i.e., changes between baseline, post, and follow-up time points) for an alpha of 0.05. In addition, if for some dependent variables, the power is insufficient to detect a change, we will use Bayesian Analysis (Fitzpatrick et al., 2016) instead of APIM/M as authors have previously recommended (Sagan and Kowalska-Musiał, 2009; Ledermann et al., 2022). All the statistical analyses will be conducted using R (R Core Team, 2021). If multiple statistical analyses (e.g., *post hoc* ANOVAs or *t*-tests) are to be conducted sequentially on the same set of dependent variables, the level of significance (alpha) will be divided by the number of tests (multiple comparisons correction) to reduce the risk of false positives (i.e., reducing Type-I errors).

## 4. Limitations

Despite the importance and innovative aspect of the study, many limitations are to be noted. First, the study is to be conducted with a Canadian English-speaking sample, which might limit the generalizability of the results to non-English speaking or non-Canadian/Western population. Repeating the study in different populations with different language, geographic and ethnic background will be highly warranted. Second, we intend to implement a brief (low dose) MBSR program, which, even has been shown to produce similar effects than the original program, might limit the effects on some specific outcomes related to interpersonal mindfulness. Third, even though mindfulness is measured with both a subjective (self-report) and objective (behavioral) measure, the use of MAAS limits the measure of mindfulness to a single dimension. Finally, mindfulness practice is only measured through self-report daily logs, which might not be accurate as participants tend to forget and might have difficulty to be objective in evaluating their own practice. In sum, most of these limitations should be addressed in future studies testing the proposed model of interpersonal mindfulness.

## 5. Significance and implications

Many studies have shown the positive effects of mindfulness training on those who engage in it, and others have shown a positive impact on interpersonal relationships, or on participants’ partners. This is the first attempt to investigate these three processes in a single study. In addition, this is the first study that grounds the study of interpersonal mindfulness in the quality of interpersonal interactions, using objective and subjective measures. Moreover, this is the first study that examines interpersonal mindfulness among unrelated (i.e., stranger) dyads. The field of interpersonal mindfulness is growing fast, however, processes of interpersonal mindfulness, that is, the mechanisms through which the positive effects of mindfulness are transferred from individuals receiving the training to those interacting with them remain unknown. Therefore, investigating the interpersonal effects of mindfulness training through the proposed model allows us to establish the theoretical and empirical basis of interpersonal mindfulness, which is crucial for future research. More specifically, this project establishes the scientific bases of the transfer of mindfulness benefits from individuals receiving mindfulness training to individuals interacting with them. In addition, the project allows to measure the effects of mindfulness meditation on prosocial processes (such as empathy, active listening, compassion toward others, quality of social emotional support) and to delineate the role of these prosocial processes in interpersonal mindfulness.

The study implements a shortened version of MBSR and MBSR is the most used MBP worldwide, increasing therefore, the external validity of the study. In addition, the longitudinal aspect of the study allows testing the long-term effects of mindfulness training on interpersonal interactions. Furthermore, the study of the effects of MBSR training on interpersonal interactions through behavior observation will reveal for which verbal and non-verbal interpersonal behaviors MBSR is effective. On the social level, delineating the active ingredients of interpersonal mindfulness and specifically the role of prosocial behaviors will allow devising and validating new and more effective interpersonal mindfulness-meditation programs, with applications in multiple fields, including parenting, teaching, counseling, physical/mental health, management, and intimate/conjugal relationships among others.

## Data availability statement

The original contributions presented in this study are included in the article/supplementary material, further inquiries can be directed to the corresponding author.

## Author contributions

BK conducted the literature review, the design of the protocol, and wrote the first draft of the manuscript. VM helped in conducting the literature review and edited the manuscript. GD, ML, and HS edited the manuscript and provided feedback. RCV provided advice on the used methodology and analyses. LA provided feedback on the manuscript. SR edited the manuscript and provided advice on the protocol. All authors contributed to the article and approved the submitted version.
